# Numerical and Experimental Extraction of Dynamic Parameters for Pyramidal Truss Core Sandwich Beams with Laminated Face Sheets

**DOI:** 10.3390/ma13225199

**Published:** 2020-11-17

**Authors:** Miroslaw Wesolowski, Mariusz Ruchwa, Eduards Skukis, Andrejs Kovalovs

**Affiliations:** 1 Department of Structural Mechanics, Faculty of Civil Engineering, Environmental and Geodetic Sciences, Koszalin University of Technology, ul. Sniadeckich 2, 75-453 Koszalin, Poland; mariusz.ruchwa@tu.koszalin.pl; 2 Institute of Materials and Structures, Riga Technical University, Kalku Street 1, LV-1658 Riga, Latvia; edskukis@gmail.com (E.S.); andrejs.kovalovs@rtu.lv (A.K.)

**Keywords:** dynamics, sandwich beam, laminates, FEM, vibration testing, damping, mode shape

## Abstract

Sandwich beams that are composed of laminated face sheets and aluminum pyramidal truss cores are considered to be essential elements of building and aerospace structures. In this paper, a methodology for the experimental and numerical analysis of such structures is presented in order to support their industrial application. The scope of the present research covers both the experimental and numerical extraction of the dynamic parameters of the sandwich beams. Vibration tests are performed while using an optical system for three-dimensional vibrations sensing. The in-plane and out-of-plane vibration modes can thus be examined. A detailed numerical model of the sandwich beam is developed, including an adhesive joint (an additional layer of material) between the parent components of the beam. The numerically predicted modal parameters (eigenfrequencies, mode shapes, modal loss factors) are comported with their corresponding experimentally-obtained values. The modal loss factors are predicted based on the strain energy method, for which a brief theoretical introduction is provided. The obtained experimental and numerical results coincide with good accuracy. The circumstances for possible model simplifications are provided depending on the solution objectives.

## 1. Introduction

The numerical modeling of various types of structures has become an indispensable part of engineering design work. The problems that are related to the analysis of structures made of metal, timber, or composite materials are often addressed through numerical analyses. Such analyses are particularly useful when a structure becomes increasingly anisotropic, which is often the case with composite structures. There is a large variability of types of composite structures. Consequently, numerical tools for analyzing composite structures’ behavior are constantly being developed and improved upon. One type of these structures is a sandwich composite with a pyramidal truss core. This particular type of composite has recently gained the attention of the scientific community due to its engineering advantages [1,2,3,4,5]. Among these advantags, they possess high specific bending stiffness, weight efficiency, and good thermal and acoustical insulation [6]. The static and dynamic behavior of sandwich structures is governed by the configurations of the parent components (face sheets and core), as well as their constituent materials’ properties. The face sheets of these structures mainly contribute to the bending stiffness, whereas the core mainly contributes to the shear stiffness. The manufacturing of such composites is realized while using adhesive joints between the core and the face sheets [7]. If the parent materials are made of metal, the joints are mostly manufactured by brazing or laser welding [8]. However, if the parent components are made of different materials, e.g., fiber-reinforced laminated face sheets and aluminum core, then the joints must be manufactured by the application of an adhesive material such as epoxy resin. For the first case, it is customary to assume that the brazed or welded joints possess similar material properties to those of the parent material and, therefore, there is no need for special modeling of the joints. For the latter case, the adhesive material’s properties differ from those of the parent components. This may result in a significant mutual displacements between them. For such a case, it is important to properly model the adhesive joint in order to capture additional effects that are associated with the mutual movements of the parent components. The current state-of-the-art regarding the modeling of sandwich beams with a pyramidal truss core is mostly governed by two approaches, namely equivalent modeling [9,10] or the finite element method (FEM) [11,12]. The first approach has usually been used for static analyses including linear and non-linear responses as well as fracture analysis. The equivalent model of the sandwich beam is mostly derived from the geometrical characteristics of the core and the stiffness parameters of parent components. Such models are suitable for analyzing full metal sandwich beams as well as different combinations of parent components, such as metal face sheets with a laminated carbon fiber composites (CFRP) truss core or CFRP face sheets with a metal truss core. However, for this kind of model, the adhesive joints’ compliance is not accounted for. For this reason, this type of structural modeling does not provide any information on the local behavior of the sandwich beams. The FEM approach has been used for either static or dynamic (vibration and crashworthiness) analyses. It allows for the detailed modeling of structures. However, this approach requires careful planning of the model development. It must start with proper assumptions made regarding the geometry, element type, material models, interactions between parts that are in contact (for the current case parent components), number of elements, type of solution, etc. The most attention is commonly given to the exact representation of the geometry of the beams, as seen in the up-to-date literature. The mutual contact between parent components is mostly defined as a tied contact (no mutual displacements between parent materials are allowed) [13] or by the introduction of a mathematical model for adhesive joints (small displacements between parent materials are allowed) [14]. However, the motivation for such approaches has been not justified, unless the parent components are made of the same material [15]. There is also a number of research studies devoted to the sandwich beams with an adhesive layer made of viscoelastic layer [16,17,18]. However, for the above cases, the viscoelastic layer was designed in order to enhance damping behaviour only, rather than the bound between the face sheets.

In the present paper, the authors aim to establish the role of the locally applied adhesive joints in the dynamic response of the sandwich beam. For this purpose a detailed three dimensional finite element model of the beam is developed. The beam is composed of laminated face sheets and the pyramidal aluminum truss core. The model accounts for a layer of the adhesive material that is placed between the parent components. The authors assume that the adhesive material behaves as an elastic solid but possess the both stiffness and dynamic properties. As a consequence, the overall beam’s compliance changes and affects the dynamic parameters of the beam. In order to model the adhesive layer a continuum hybrid solid-shell element is used which allows considerably reducing the total number of finite elements. The dynamic parameters of interest are eigenfrequencies, mode shapes, and modal loss factors. The significance of the adhesive joint modeling is verified by developing two numerical models of the sandwich beam with a different approach for the joint definition. The first approach accounts for additional layers of material that are placed between parent components. In the second approach, the joint is defined as a tided contact with no mutual displacement between parent components allowed. In addition, the sensitivity of the beam’s eigenfrequencies to the stiffness variation of the adhesive material is presented. The most appropriate model will be used for the modal parameters prediction. The modal loss factors are predicted based on the strain energy method, for which a theoretical background is presented. The scope of the present investigation is then expanded to a series of experimental vibration tests on a real sandwich beam samples. The vibration tests are meant to validate the correctness of the developed numerical models. For the vibration tests, a system of three laser vibrometers is used, which are capable of sensing vibrations in three mutually perpendicular directions. The in-plane and out-of-plane vibration modes of the sandwich beam can be therefore investigated, which is seldom reported in the up to date literature. The results presented in the current paper will contribute to field of sandwich structures dynamics by defining the extent and circumstances for the possible model simplifications in terms of adhesive layer modeling. The above will be achieved by comparing the numerical and experimental analyses.

## 2. Materials and Methods

### 2.1. Samples

**Figure 1:**
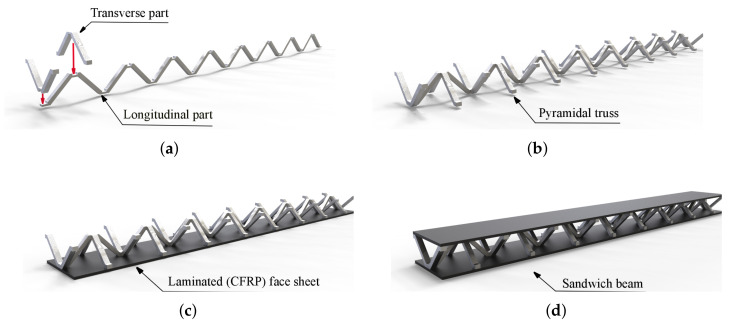
Fabrication process of a sandwich beam: (**a**,**b**) aluminum pyramidal truss core assembly; (**c**,**d**) a sandwich beam assembly with laminated face sheets and pyramidal core. CFRP: carbon fiber reinforced plastic.

**Figure 2:**
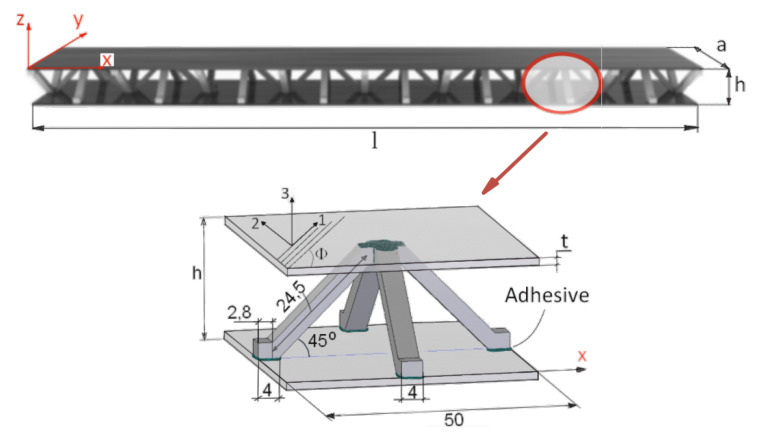
Characteristic dimensions of the sandwich beam.

The objects of the current research are sandwich composite beams. The sandwich beams were composed of two parent components: (1) upper and lower face sheets made of laminated carbon fiber reinforced plastic (CFRP); and, (2) a pyramidal truss core made of the aluminum alloy PA6 (Figure 1d). A global Cartesian coordinate system (x,y,z) was located in the corner of the beam, with the *z* axis normal to the face layer (Figure 2). For the CRFP laminated face sheets, a lamina principal coordinate system (1,2,3) was defined with the direction 1 along the fibers of the tape, 2 transverse to this direction, and 3 through the thickness direction. A lamination angle (Φ) of the fibers was defined between the *x*-axis and the 1-axis (Figure 2). The face sheets were cut out of a long single-layer unidirectional CRFP tape. The tape was manufactured by means of a pultrusion process. The pyramidal truss core was assembled from separate pieces (longitudinal and transverse), which were cut out of an PA6 aluminum plate by means of water-jet cutting. The PA6 is an aluminum alloy possessing relatively high hardness and good corrosion resistance. The PA6 is easy to machining and press. However, it not well-suited for welding, but it can be processed at low and moderate temperature levels. For this reason, adhesive joining is recommended for PA6. Thus, the aluminum pieces were bonded together with a thermosetting epoxy adhesive to form a continuous pyramidal truss core (Figure 1a,b). Figure 2 provides the geometry of a single cell of the truss. The parent components were bonded together to form a sandwich beam using the same thermosetting epoxy adhesive as that used for the core assembly (Figure 1c,d). The epoxy adhesive was applied locally at the contact spots between the sheets and the core (Figure 2). Five samples were manufactured for experimental investigation with characteristic dimensions, as given in Table 1.

### 2.2. Numerical Model of Sandwich Beam

The finite element method (FEM) was used in order to develop a numerical model of the sandwich beam. The Simulia/Abaqus software was used as a pre- and post-processor. The FEM model was assumed to be an assembly of three components (instances), namely laminated face sheets, aluminum pyramidal truss core, and adhesive material. Table 2 shows the elastic material’s components’ properties. The dynamic material’s components’ properties are given in Table 3. Both the elastic and dynamic materials’ properties were established by means of an inverse technique, as described in [19,20], respectively.

#### 2.2.1. Modeling of Laminated Face Sheets

The laminated tape used for the face sheets was considered to be a thin and narrow plate. For the case of thin plates or shells, it is customary to use shell elements [21,22,23,24]. Furthermore, as the laminated sheets were produced as a one-layer tape, a single-layer shell element can be used. It was also assumed that the first order shear deformation theory (FSDT) possesses satisfactory strain-displacement relations. To fulfill the above assumptions, the S4R shell element with an orthotropic elastic material model was used for the development of the face sheets of the sandwich beam. The lamina’s elastic and dynamic material properties were associated with the corresponding axes (1,2,3) of the lamina’s principal coordinate system.

#### 2.2.2. Modeling of an Aluminum Pyramidal Core

**Figure 3:**
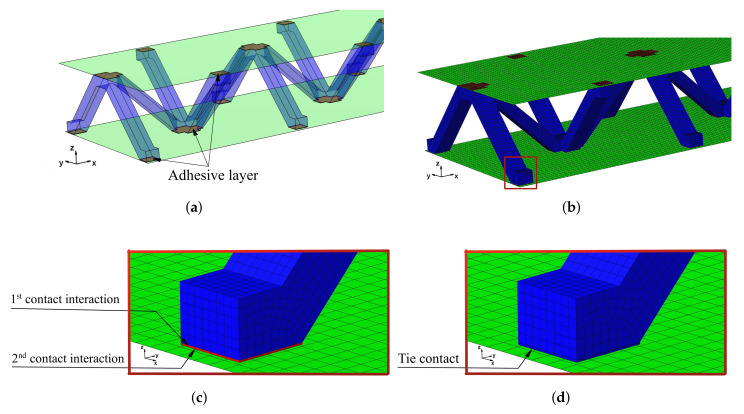
Development procedure of the numerical model: (**a**) parent materials assembly; (**b**) associated mesh; (**c**) joint modeling with the adhesive layer; (**d**) joint modeling without the adhesive layer.

The aluminum pyramidal core was considered to be a solid 3D body. No adhesive connections between the longitudinal and transverse parts of the core were modeled. In order to discretize the 3D body, the linear brick elements C3D8R were used with an isotropic elastic material model (Figure 3b).

#### 2.2.3. Modeling of an Adhesive Layer

An adhesive joint between the pyramidal truss and face sheets has the form of a thin layer of an additional material. Consequently, the shell elements were selected for the discretization of the adhesive layer. For this purpose, a continuum hybrid solid-shell elements SC8R was used. The SC8R elements discretize an entire three-dimensional body. They only possess displacement degrees of freedom. From a modeling point of view, the SC8R elements look like three-dimensional continuum solids, but they have kinematic and constitutive behavior that is similar to conventional shell elements. The advantage of the SC8R elements is that they exhibit fast element convergence along the thickness direction. For the adhesive layer, one element in the thickness direction is sufficient. The SC8R elements also allow for two-sided contact with changes in their thickness, which makes them more suitable for the modeling of contact interactions. The adhesive layers were only modeled in the spots where contact between the truss and face sheets existed (adhesive joints).

#### 2.2.4. Model Assembly and Solution Method

The model of the sandwich beam was assembled from the separate instances based on the contact definition. The length l=386.1 mm of the beam was set as the mean value of the beams’ length given in Table 1. The rest of the beam’s dimensions were set as: a=50.0 mm, h=27.8 mm and t=1.4 mm. The lamination angle was Φ = 0 [deg]. The thickness of the adhesive layers was set as 0.1 mm. Two models were developed for a comparative purpose. The first model (hereafter FEM(I)) accounts for the additional material that was placed between parent components for the adhesive joint modeling. The assembly of the sandwich beam with adhesive layers was performed by means of the two tie contact interactions at each adhesive joint. The first contact was defined as between the inner surface of the adhesive layer and the outer surface of the aluminum pyramidal truss core. The second contact was defined as between the outer surface of the adhesive layer and the inner surface of the laminated face sheets. The above contact modeling approach resulted in an adhesive joint compliance that is dependent on the mechanical properties of the adhesive material. For the second model (hereafter FEM(II)), the parent components were connected while using one tie contact at each joint. The contact was defined as between the inner surface of the laminated face sheets and the outer surface of the aluminum core. The model development procedure is schematically given in Figure 3.

For the current study, the solution of the eigenvalue problem for undamped free vibrations was calculated, as follows:

(1) $$\left(K-\omega_{n}^{2}M\right)\Theta_{n}=0$$

where K and M are the stiffness and mass matrices of the sandwich beam, respectively; $\Theta_{n}$ are the eigenvectors (mode shapes) of the corresponding eigenvalues $\omega_{n}=2\pi f_{n}$, where $f_{n}$ are eigenfrequencies. The modal analysis with the Lanczos mode-extraction method was applied in order to determine eigenvalues and corresponding eigenvectors. For each shell element, five integration points were selected, from which the strain and stress components were stored. A convergence study was performed for the numerical model and the final number of elements for each parent component was established (Table 4). In addition, the sensitivity of the beam’s eigenfrequencies to the stiffness variation of the adhesive material was performed. For this purpose, the adhesive’s Young’s modulus was set within the range of 1.46 GPa ± 50%.

### 2.3. Damping Model

The applied damping model is based on the modal strain energy principle. The proposed damping model for fibrous composites was developed by the authors of [25,26]. The method introduces the specific damping capacity (SDC) ${}^{s}\Psi_{n}$ as a measure of an energy loss. The SDC is given as the ratio of the total dissipated energy $\Delta^{s}U_{n}$ to the maximum strain energy ${}^{s}U_{n}$ that is stored in the structure during a stress cycle at the nth mode of vibration:

(2) $${}^{s}\Psi_{n}=\frac{\Delta^{s}U_{n}}{{}^{s}U_{n}}$$

The SDC is related to the modal loss factor ${}^{s}\eta_{n}$ as:

(3) $${}^{s}\Psi_{n}=2\pi^{s}\eta_{n}$$

The proposed damping model was combined with the FEM analysis for the modal loss factors prediction of the sandwich beam.

#### 2.3.1. Strain Energy

We propose expressing the modal loss factor of the sandwich beam as a sum of the damping fractions from the individual parent components and adhesive material. The total strain energy stored for a particular mode shape of a sandwich beam can be written as (the mode number was dropped for notational convenience):

(4) $${}^{s}U={}^{l}U+{}^{c}U+{}^{a}U$$

where ${}^{l}U$, ${}^{c}U$, and ${}^{a}U$ are the strain energies that are stored in the two laminated face sheets, aluminum pyramidal truss core, and adhesive material, respectively.

The strain energy that is stored in a single finite element *e* of the laminated face sheets is the sum of the energies stored in the principal lamina directions:

(5) $${}^{l}U^{e}={}^{l}U_{11}^{e}+{}^{l}U_{22}^{e}+{}^{l}U_{12}^{e}$$

The elements’ energies that are given in Equation (5) are related to the strain and stress components as:

(6) $${}^{l}U_{ij}^{e}=\frac{1}{2}\cdot \varepsilon_{ij}^{e}\cdot \sigma_{ij}^{e}\cdot V^{e}$$

where ${}^{l}U_{ij}^{e}$ is the strain energy stored in element *e* in the principal lamina directions for ij=11,22,12, (with 11 along the fibers’ direction, 22 transverse to the fibers’ direction and 12 in-plane); $\varepsilon_{ij}^{e}$ and $\sigma_{ij}^{e}$ are the strain and stress components, respectively, and $V^{e}$ is the volume of the element *e*.

Thus, the total strain energy stored in the laminated face sheets made of *M* elements is:

(7) $${}^{l}U=\sum_{e=1}^{M}{}^{l}U_{11}^{e}+\sum_{e=1}^{M}{}^{l}U_{22}^{e}+\sum_{e=1}^{M}{}^{l}U_{12}^{e}$$

The truss core is made of isotropic aluminum material and, therefore, the strain energy ${}^{c}U^{p}$ that is stored in the finite element *p* of the pyramidal truss core is obtained from the strain and stress tensor calculated in the global (x,y,z) coordinate system. Thus, the total strain energy that is stored in the core made of *J* elements is:

(8) $${}^{c}U=\sum_{p=1}^{J}{}^{c}U^{p}$$

The adhesive material is assumed to behave as an isotropic material. The strain energy ${}^{a}U^{r}$ stored in the finite element *r* of the adhesive material is obtained in a similar manner to the truss energies in the global (x,y,z) coordinate system. Thus, the total strain energy that is stored in the adhesive material made of *R* elements is:

(9) $${}^{a}U=\sum_{r=1}^{R}{}^{a}U^{r}$$

#### 2.3.2. Dissipated Energy

The energy that is dissipated by the finite element *e* of the laminated face sheets is given as:

(10) $$\Delta {}^{l}U^{e}=\psi_{11}{}^{l}U_{11}^{e}+\psi_{22}{}^{l}U_{22}^{e}+\psi_{12}{}^{l}U_{12}^{e}$$

where $\psi_{ij}$ (ij=11,22,12) are the specific damping capacity coefficients (SDC coefficients) of the material used for the laminated face sheets. The total energy that is dissipated by the laminated face sheets is:

(11) $$\Delta {}^{l}U=\sum_{e=1}^{M}\Delta {}^{l}U^{e}$$

The energy dissipated by element *p* of the aluminum truss core is given as:

(12) $$\Delta {}^{c}U^{p}=\psi_{c}{}^{c}U^{p}$$

where $\psi_{c}$ is the SDC coefficients of the material used for the pyramidal truss core. The total energy dissipated by the core is:

(13) $$\Delta {}^{c}U=\sum_{p=1}^{J}\Delta {}^{c}U^{p}$$

Accordingly, the energy dissipated by element *r* of the adhesive material is given as:

(14) $$\Delta {}^{a}U^{r}=\psi_{a}{}^{a}U^{r}$$

where $\psi_{a}$ is the SDC coefficients of the adhesive material. The total energy that is dissipated by all of the adhesive joints is:

(15) $$\Delta {}^{a}U=\sum_{r=1}^{R}\Delta {}^{a}U^{r}$$

Thus, the total energy dissipated by the sandwich beam is given as:

(16) $$\Delta {}^{s}U=\Delta {}^{c}U+\Delta {}^{l}U+\Delta {}^{a}U$$

#### 2.3.3. Damping Model Validation

The proposed damping model was validated based on the results that are given in [26], where the SDC coefficients were predicted for a square laminated plate. The model of the plate was reproduced using Simulia/Abaqus software and the frequency analysis was performed in order to extract the mode shapes of the plate. The S4R elements were used for modeling purposes. The total number of elements was 144. The plate thickness was t=1.58 mm with the lamination angle set as Φ = 0 [deg]. Side length of the plate was l=178 mm. Free boundary conditions were assumed for all edges of the plate. The engineering constants used in the analysis were as follows: $E_{1}=172.7$ GPa, $E_{2}=7.20$ GPa, $G_{12}=3.76$ GPa, $\nu_{12}=0.3$. The dynamic parameters were: $\psi_{11}=0.45\%$, $\psi_{22}=4.22\%$, $\psi_{12}=7.05\%$, ρ=1566.0 kg/m${}^{3}$. As reported in Table 5, the results coincided well with the average difference 2.26%.

Having established and validated the damping model, the procedure for modal loss factor prediction was applied for a selected number of vibration modes of the sandwich beam model. All of the information required for the calculations was extracted from the results of the frequency analysis that were provided by Simulia/Abaqus software. For a more detailed description of the modal loss factor prediction procedure the reader is encouraged to refer to [20,27].

### 2.4. Experimental Set-Up

**Figure 4:**
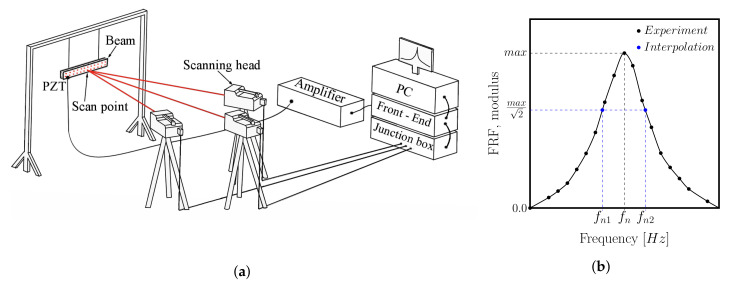
Experimental assessment of a modal damping: (**a**) three-dimensional (3D) laser vibrometer set-up; (**b**) half-bandwidth method for modal loss factor extraction.

In the current research, a non-contact method for vibration sensing was applied while using the scanning laser vibrometer POLYTEC PSV-500-3D. The PSV-500-3D system operates on the Doppler principle by measuring the frequency shift of the back-scattered laser light from the vibrating object. For this purpose, a velocity decoder is used (VD-07). The whole system is built of three independent scanning heads, a front end, a junction box, a PC station, and a power amplifier (Figure 4a). The application of the three scanning heads allows for measuring three mutually orthogonal displacement components at a measurement point. This gives the opportunity for extracting out-of-plane as well as in-plane modes of vibration. The out-of-plane vibration modes are those for which the deflection is measured only on the *z* direction (normal to the plane of the face sheet). The in-plane modes are those for which the deflection is measured in the xy plane of the beams (parallel to the plane of the face sheet). As a result, the in-plane mode is a magnitude of the deflections in the *x* and *y* directions. The PSV-500-3D is equipped with an internal signal generator that drives the test object into vibrations through the piezoelectric disc actuator (PZT). The PSV-500-3D system is capable of a scanning procedure (multipoint measurements). This offers the possibility for storing an average frequency response function (FRF) and it provides high-quality operational shapes of a specimen. The obtained FRFs are then used for the extraction of the modal loss factors.

#### Modal Loss Factors Extraction

The extraction of experimental modal loss factors was based on a frequency domain single degree of freedom (SDOF) approach. The SDOF approach imposes a step-by-step sequential analysis in the vicinity of each peak from the modulus plot of the FRF (Figure 4b). For this purpose, a peak-amplitude method can be used [28]. According to this method, the modal loss factor that is extracted from the experimental tests ${}^{s}\eta_{n}^{EXP}$ for a particular mode (or resonance peak) *n* is calculated as:

(17) $${}^{s}\eta_{n}^{EXP}=\frac{f_{n2}^{EXP}-f_{n1}^{EXP}}{f_{n}^{EXP}}$$

where *n* stands for a mode number; $f_{n1}$ and $f_{n2}$ are frequencies whose values were interpolated from the modulus plot of the FRF (Figure 4b). For this purpose, the spline interpolation method was implemented using the Matlab subroutine.

## 3. Results

**Figure 5:**
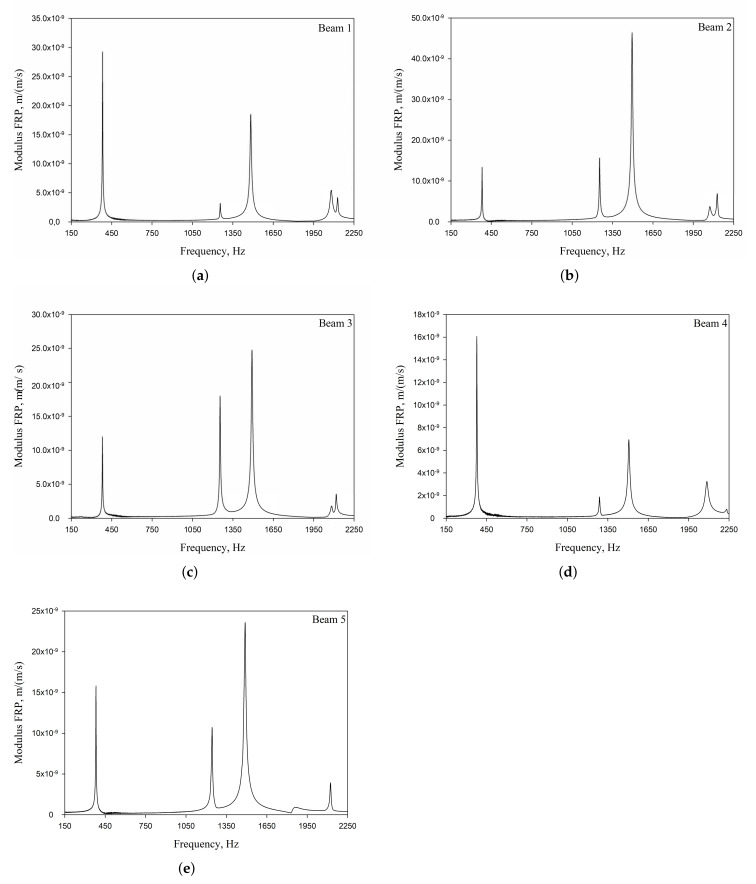
Experimental Frequency Response Functions (FRFs) of the sandwich beams: (**a**) Beam 1; (**b**) Beam 2; (**c**) Beam 3; (**d**) Beam 4; (**e**) Beam 5.

**Figure 6:**
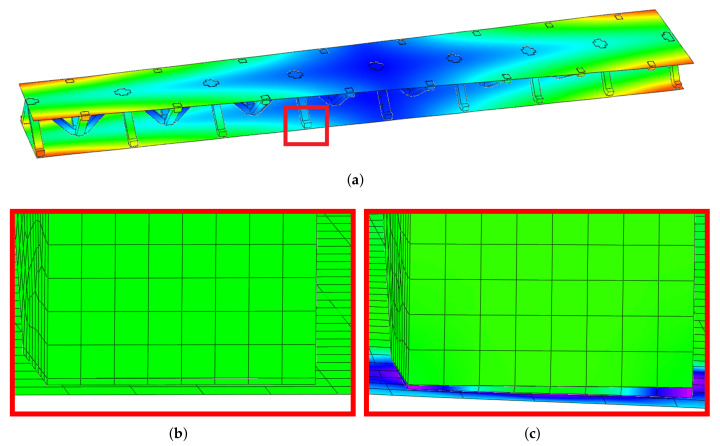
Numerical results: (**a**) Example of the deformed mode shape (mode 1); (**b**) undeformed state of the adhesive layer; (**c**) deformed state of the adhesive layer.

**Figure 7:**
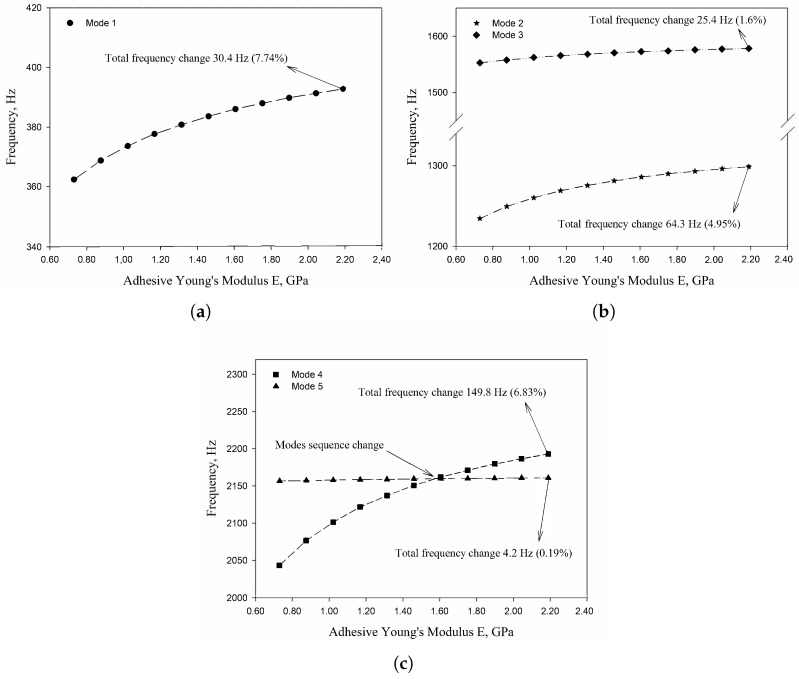
Sensitivity study of the eigenfrequency change due to the adhesive E modulus variation: (**a**) Mode 1; (**b**) Mode 2 and 3; (**c**) Mode 4 and 5.

**Figure 8:**
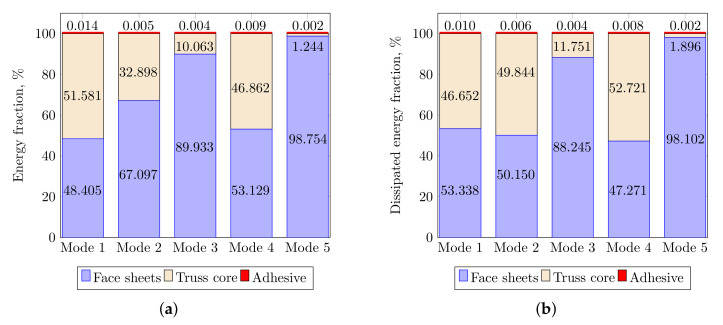
Finite element method (FEM) results: (**a**) strain energy; (**b**) dissipated energy.

The vibration tests were performed for the five sandwich beams. The excitation range was set as 0 ÷ 2250 Hz. The beams were driven to vibrations by means of a thin piezoelectric disc (PZT) with a mass of 3 g. Each beam was hung on a very thin cotton threads in order to simulate the free boundary conditions. The frequency response functions (FRFs) were stored for each beam. Figure 5 provides the plots of the modulus FRFs. Five resonance peaks emerged from the FRFs. The corresponding mode shapes for these resonances are given in Table 6. Four modes were the out-of-plane modes (modes 1, 2, 3, 4) and one mode was the in-plane bending mode (mode 5). The FRFs data of each beam were exported to MATLAB software and the modal loss factors were estimated. Table 7 presents all of the experimental results for the five tested sandwich beams. It must be pointed out that significant result discrepancies were noticed for beams 4 and 5. Additionally, the FRFs of those beams exhibited some distortions, especially for higher modes (Figure 5d,e). Therefore, it is a common practice to neglect such results. Hence, for the calculating of average values, only the results for beams 1, 2, and 3 were used. The solution of the frequency analysis of the developed models (FEM(I) and FEM(II)) was run. Free boundary conditions were assumed for all the edges of the sandwich beam. The frequency range was defined as 0 ÷ 2250 Hz. The analysis resulted in five modes of vibrations for each model (Table 6). It must be noted that, in the case of the model FEM(II), there was a change in the sequence of the mode shapes’ appearance. In addition, a considerably higher discrepancy between the model results and experimental results was noticed for the FEM(II) model. What is more, the mutual displacement of the parent components was captured form the solution of the model FEM(I) due to non-uniform deformation of the adhesive layer (Figure 6). For that reason, the FEM(I) model was selected for modal damping predictions. The FEM(I) model was also used for the sensitivity of the eigenfrequencies due to the E modulus variation of the adhesive material. Figure 7 provides the plots of the eigenfrequencies changes. The elements’ strain and stress values for each mode of vibration as well as the elements’ volumes were extracted from the obtained results. The modal loss factors were calculated based on those data and the material properties that are given in Table 2 and Table 3. The fractions of the total strain energy, as well as the dissipated energy associated with each parent material, were also calculated (Figure 8). Table 8 and Table 9 provide the comparisons of the experimental results and the numerical predictions.

## 4. Discussion

The research that is presented in the current paper covers experimental and numerical dynamic analyses of sandwich beams. The real sandwich beam samples were composed of laminated face sheets and an aluminum pyramidal truss core. The above were assembled together by means of an adhesive joint using epoxy resin. An additional layer of a material was also included in the numerical model of the beam, in which isotropic material was placed between parent components at their contact points. A simplified approach for the joint modeling was also presented while using the tie contact definition.

The experimental vibration tests gave satisfactory results.The application of the optical 3D scanning system POLYTEC PSV-500-3D allowed for extracting high-quality FRFs and corresponding mode shapes. For all of the modes, the modal loss factor was successfully calculated based on the modulus plot of the FRF. The non-invasive vibration sensing considerably reduced the influence of any additional measurement instrumentation on the damping values. An important aspect of the experimental procedure was the extraction of the in-plane mode of vibration. Note that the in-plane mode as well as the out-of-plane modes were extracted during one vibration test. From the five samples tested experimentally, the results of three beams (beams 1, 2, 3) agreed well. For two beams (beam 4, 5), considerable result discrepancies were noticed. In the authors’ opinion, this situation was due to the samples’ manufacturing quality, rather than due to the equipment used or the measurement procedure. Thus, average values were calculated based on the experimental results that were extracted from three beams. These values were then used for comparison purposes with the numerical results.

Two FEM models of the sandwich beams were developed for the numerical prediction of the modal parameters. One model (FEM(I)) accounted for a layer of the adhesive material in order to connect parent materials (laminated face sheets and the aluminum pyramidal truss core). The second model (FEM(II)) was simplified by the elimination of the adhesive layer. The parent materials were connected by tie contact definitions. Both of the models were subjected to frequency analysis in order to extract the eigenfrequencies and corresponding mode shapes. Significant result discrepancies were noticed between the developed models as well as versus the experimental results, as given in Table 8. The eigenfrequencies for the FEM(II) model were overestimated, which is a consequence of higher overall beams’ compliance values due to the tie contact definitions. The results given for the FEM(I) model were far more consistent with the experimental results. This was a consequence of the beam’s compliance reduction due the deformation of the adhesive material. An important issue is that a change in the mode shape’s appearance was noticed for the FEM(II) model. As given in Table 6, the last two modes interchanged. Such a situation is not acceptable if the dynamic behaviour of a structure is the main focus for the designers, or if the model is intended to be used for optimization purposes. The phenomenon of the modes sequence change was also captured from the sensitivity study. As given in Figure 7c, the increase of the adhesive modulus considerably changes the eigenfrequency of the mode 4, but has negligible effect on the mode 5. As a result, the two values cross at a point were the modes sequence changes. The increase of the adhesive modulus brings all the eigenfrequencies values close to the FEM(II) solution. However the rate of change is not the same for all of the considered modes. As for the modal loss factor predictions, the proposed procedure for the modal damping prediction was successfully validated with the available literature. The stress and strain components calculated for the FEM(I) model were then used for the prediction of the modal loss factors of the sandwich beams. The strain energy for a single shell finite element of the laminated face sheets was calculated while using Equation (6). It must be pointed out, that Equation (6) was applied for each integration point throughout the element thickness. The element’s strain energy was taken as an average value from all integration points. For that reason, it is recommended to define at least five integration points throughout the lamina thickness. The fractions of the energies stored in each parent component as well as the fractions of the dissipated energies were calculated and are given in Figure 8. Following the results, the contribution of the adhesive layer to the overall beam’s strain energy is negligible for each mode of vibration. Accordingly, the fraction of the dissipated energy due to the material of the adhesive layer is also negligible. Hence, the material that is used for the adhesive joint (epoxy adhesive in the present study) does not considerably contribute to the total beam’s energy dissipation. Nevertheless, as given in Table 9, a good agreement was achieved between the numerical prediction and the experimental results. The average difference of the results was found to be 11.58%. However, the predicted modal loss factor for mode no. 4 differed significantly from the experimental results. In the authors’ opinion, this might be the reason for closely spaced modes (see the FRFs of the beams in Figure 5). For such a case, a more sophisticated approach for modal parameters extraction shall be used. The average results’ difference could also be reduced if the measurements were performed in a vacuum chamber. The surrounding air may have significantly affected the damping assessment, as the examined sandwich beams were open cell type structures.

## 5. Conclusions

The experimental and numerical analyses that are presented in the current paper allow to us draw the following conclusions. Regarding the eigenfrequencies and mode shape predictions, the modeling of the adhesive layer is fully justified. The numerical predictions coincide well with the experimental results. Care shall be taken for the closely spaced vibration modes, as the lack of the adhesive material at the joints may affect the modes’ appearance sequence. Little effect of the adhesive layer was found on the modal loss factors predictions. However, more types of the adhesive materials shall be investigated, e.g., a viscelastic layer, in order to draw a final conclusion on this matter. The 3D system for the optical vibrations’ sensing demonstrated its ability to very efficiently extract both the out-of-plane and in-plane vibration modes. 

## Figures and Tables

**Table 1:** Test samples.

Sample	*l* [mm]	*a* [mm]	*h* [mm]	*t* [mm]	Φ [mm]
Beam 1	386.3	50.0	27.75	1.4	0
Beam 2	385.9	50.0	27.80	1.4	0
Beam 3	386.2	50.0	27.75	1.4	0
Beam 4	386.5	50.0	27.80	1.4	0
Beam 5	386.6	50.0	27.75	1.4	0

**Table 2:** Elastic properties of the parent materials.

Parent Component	Material	E [GPa]	E${}_{1}$ [GPa]	E${}_{2}$ [GPa]	G${}_{12}$ [GPa]	G${}_{13}$ [GPa]	G${}_{23}$ [GPa]	$\nu_{12}$	ν
Face sheet	CFRP	-	142.2	10.5	7.8	7.8	3.8	0.3	-
Core	PA6	66.2	-	-	-	-	-	-	0.25
Adhesive	Epoxy	1.46	-	-	-	-	-	-	0.25

**Table 3:** Dynamic properties of the parent materials.

Parent Component	Material	$\psi_{11}\left[\%\right]$	$\psi_{22}\left[\%\right]$	$\psi_{12}\left[\%\right]$	$\psi_{c}\left[\%\right]$	$\psi_{a}\left[\%\right]$	ρ [kg/m${}^{3}$]
Face sheet	CFRP	0.63	8.30	5.45	-	-	1590.0
Core	PA6	-	-	-	3.85	-	2700.0
Adhesive	Epoxy	-	-	-	-	3.00	1200.0

**Table 4:** Numerical model statistics.

Parent Component	Element Type	Number of Elements	Material Model
Face sheets	S4R	38,600	Orthotropic, elastic
Core	SC8R	154,832	Isotropic, elastic
Adhesive	C3D8R	5376	Isotropic, elastic
	Total	198,808		

**Table 5:** The predicted values of the specific damping capacity (SDC) coefficients for square laminated plate vs. [26].

Mode	FEM Present	FEM [26]	Difference, %
1	0.0690	0.0676	2.03
2	0.0422	0.0428	1.42
3	0.0607	0.0589	2.97
4	0.0422	0.0413	2.13
5	0.0526	0.0511	2.85
6	0.0046	0.0047	2.17
		**Average**	**2.26**

**Table 6:** Experimental and numerical mode shapes of the sandwich beam comparison.

Mode (n)	Experiment (Beam 1)	FEM (I)	FEM (II)
1	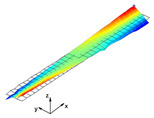	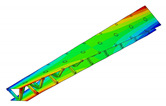	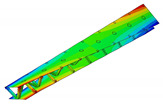
2	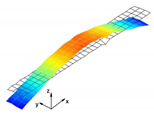	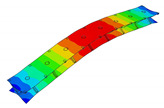	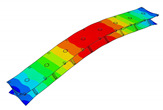
3	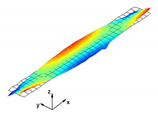	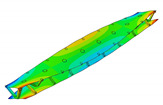	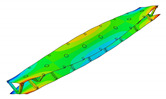
4	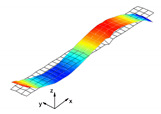	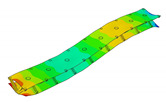	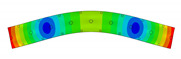
5	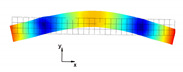	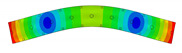	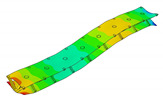

**Table 7:** Experimental results.

$\begin{array}{c} \mathrm{Mode}\\ \left(n\right) \end{array}$	Mode Type	$f_{n}^{\mathrm{EXP}},\mathrm{Hz}$	Beam 1	Beam 2	Beam 3	Beam 4	Beam 5	Average *
		${}^{s}\eta_{n}^{\mathrm{EXP}},\%$						
1	*Out-of-plane,*	$f_{1}^{EXP}$	382.60	381.20	381.10	377.70	381.80	381.65
	*(twisting)*	${}^{s}\eta_{1}^{EXP}$	0.661	0.668	0.703	0.806	0.688	0.677
2	*Out-of-plane,*	$f_{2}^{EXP}$	1256.60	1253.50	1254.60	1288.60	1243.60	1254.90
	*(bending)*	${}^{s}\eta_{2}^{EXP}$	0.365	0.363	0.367	0.454	0.533	0.365
3	*Out-of-plane,*	$f_{3}^{EXP}$	1482.40	1495.10	1492.00	1506.00	1489.00	1489.85
	*(twisting)*	${}^{s}\eta_{3}^{EXP}$	0.593	0.576	0.591	0.767	0.803	0.587
4	*Out-of-plane,*	$f_{4}^{EXP}$	2081.50	2072.50	2082.60	2085.00	1866.00	2078.85
	*(bending)*	${}^{s}\eta_{4}^{EXP}$	0.757	0.745	0.669	0.992	0.459	0.724
5	*In-plane,*	$f_{5}^{EXP}$	2128.60	2127.60	2117.80	2232.10	2122.80	2124.65
	*(bending)*	${}^{s}\eta_{5}^{EXP}$	0.383	0.363	0.336	0.836	0.354	0.361

* Average value calculated for beams 1,2,3.

**Table 8:** Experimental and numerical results’ comparison—frequencies.

Mode (n)	$f_{n}^{\mathrm{EXP}}$, Hz *	$f_{n}^{\mathrm{FEM}(I)}$, Hz	$f_{n}^{\mathrm{FEM}(\mathrm{II})}$, Hz	Δ FEM(I), %	Δ FEM(II), %
1	381.65	383.65	419.65	0.52	9.96
2	1254.9	1281.20	1328.80	2.10	5.89
3	1489.85	1570.50	1595.20	5.41	7.07
4	2078.85	2150.70	2280.60	3.46	9.69
5	2124.65	2159.50	2169.20	1.64	2.13
**Average**				**2.63**	**6.83**

* Average values from Table 7.

**Table 9:** Experimental and numerical results’ comparison—modal loss factors.

Mode (n)	${}^{s}\eta_{n}^{\mathrm{EXP}}$, % *	${}^{s}\eta_{n}^{\mathrm{FEM}(I)}$, %	Δ, %
1	0.677	0.673	0.59
2	0.365	0.404	10.68
3	0.587	0.525	10.56
4	0.724	0.545	24.72
5	0.361	0.402	11.36
**Average**			**11.58**

* Average values from Table 7.
